# The global seroprevalence of *Toxoplasma gondii* infection in workers occupationally exposed to animals (1972–2023): a systematic review and meta-analysis

**DOI:** 10.1080/01652176.2024.2396577

**Published:** 2024-08-29

**Authors:** Abdullah Mohammed, Musa Ahmed, Nasir Ibrahim

**Affiliations:** a Department of Biomedical Sciences, University of Gadarif, Gadarif, Sudan; b Department of Veterinary Surgery, AL-Salam University, West Kordofan, Sudan; c Department of Biology, Imam Mohammed Ibn Saud Islamic University, Riyadh, Saudi Arabia

**Keywords:** *Toxoplasma gondii*, seroprevalence, workers occupational exposure to animals, systematic review, meta-analysis, zoonosis

## Abstract

*Toxoplasma gondii*, a ubiquitous zoonotic parasite infecting warm-blooded animals, poses a significant health threat to workers with occupational animal exposure (WOEA) due to their frequent contact with potential reservoirs. Existing data on *T. gondii* seroprevalence in the WOEA exhibits substantial global variation. This systematic review and meta-analysis, adhering to PRISMA guidelines, aimed to quantify the global seroprevalence of *T. gondii* infection among WOEA over the past five decades (1972–2023). We identified 66 eligible studies through a comprehensive search strategy encompassing English publications, with a total sample size of 15,279. A random-effects model with the Freeman-Tukey transformation in STATA v16.0 accounted for the high heterogeneity observed. We estimated the pooled global seroprevalence of *T. gondii* infection in WOEA at 41% (95% CI: 36–47%). Subgroup analyses revealed significant variations by gender: males (63%) vs. females (37%) (*p* < 0.05), occupation: non-livestock workers (54%), livestock workers (47%), slaughterhouse workers (44%), and veterinary personnel (27%) (*p* < 0.05). Geographic trends showed the highest prevalence in Africa (51%), followed by South America (49%), Europe (47%), Australia (43%), Asia (36%), and North America (23%; *p* < 0.05). Lower prevalence was observed in high-income (39%) and upper-middle-income (38%) countries compared to lower-middle-income (44%) and low-income (48%) countries (*p* < 0.05). This analysis underscores the high global seroprevalence of *T. gondii* in the WOEA, highlighting the need for targeted interventions in this high-risk population.

## 1. Introduction

*Toxoplasma gondii* is an obligate intracellular parasite that infects virtually all warm-blooded animals, including humans (Tenter et al. 2000). Infection with *T. gondii* can cause a disease called toxoplasmosis, which can be severe and even fatal in some cases, particularly in neonates and immunocompromised individuals (Saadatnia and Golkar 2012; Wang et al. 2017). Toxoplasmosis is a major public health concern worldwide, with approximately 2 billion people infected (Rahmanian et al. 2020). The majority of infected people are asymptomatic, but some may experience mild flu-like symptoms. In severe cases, toxoplasmosis can cause a variety of complications, including encephalitis, retinitis, and seizures (Saadatnia and Golkar 2012).

Workers occupationally exposed to animals (WOEA), such as animal breeders, hunters, butchers, livestock workers, meatpacking workers, slaughterhouse workers, and veterinary personnel, are at heightened risk of *T. gondii* infection, particularly if they do not use appropriate personal protective equipment (PPE) (Wright et al. 2008; Odo et al. 2015; Habib and Alshehhi 2021). This is because many animals, including cattle, sheep, goats, camels, pigs, and chickens, can harbor the parasite (Belluco et al. 2016; Abdullah et al. 2022). WOEA can be exposed to the parasite through direct contact with infected animals or their bodily fluids, indirect contact with contaminated surfaces or objects, or accidental ingestion of raw or undercooked meat (Tenter et al. 2000).

The prevalence of *T. gondii* infection in WOEA varies widely around the world, ranging from 4.8% in a study from the United States (Rosypal et al. 2015) to 96.3% in a study from Ghana (Abu et al. 2015). This is likely due to a number of factors, including the type of animals that workers are exposed to, the level of hygiene in the workplace, and the prevalence of *T. gondii* infection in the animal population.

The global seroprevalence of *T. gondii* infection in WOEA is not well known. To date, there has been no systematic review or meta-analysis that has investigated the global seroprevalence of *T. gondii* infection in WOEA. The aim of this systematic review and meta-analysis is to estimate the global seroprevalence of *T. gondii* infection in WOEA during the last five decades, from 1972 to 2023. The findings of this review will provide valuable information to policymakers and healthcare professionals about the burden of *T. gondii* infection in WOEA.

## 2. Materials and methods

### 2.1. Study design and protocol registration

This meta-analysis adhered to the Preferred Reporting Items for Systematic Reviews and Meta-Analyses (PRISMA) guidelines (Page et al. 2021). The study protocol was prospectively registered in the International Prospective Register of Systematic Reviews (No. CRD42023412590).

### 2.2. Eligibility criteria

The full eligibility criteria used to determine studies for inclusion in the current analysis are displayed in Table 1. In addition to the criteria listed in the table, reviews, letters, editorials, and studies without full-text availability or that did not report the information needed to meet the eligibility criteria were excluded. Furthermore, studies with missing information were excluded after the primary investigator (AM) attempted to contact the corresponding author(s) *via* email and/or ResearchGate personal pages to obtain the original data but failed to obtain the missing information within 4 weeks.

**Table 1.** Study inclusion/exclusion criteria.

Inclusion	Exclusion
Studies that reported the seroprevalence of *T. gondii* infection in workers occupationally exposed to animals, including those in the following groups: livestock workers, non-livestock workers, slaughterhouse workers, fishermen, and veterinary personnel in any part of the world	Studies that did not report the seroprevalence of *T. gondii* infection in workers occupationally exposed to animals or reported it in other groups
All observational studies	Studies that were not observational in nature
Use of a validated serological test to diagnose *T. gondii* infection as described by Khan and Noordin (2020)	Studies that did not use a validated serological test to diagnose *T. gondii* infection
In English	Language limitation: Not in English
Full text of publication obtained	Full text unavailable
Studies that provided sufficient information on sample size, sampling location, or sampling period	Studies that did not provide sufficient information on sample size, sampling location, or sampling period
	Other

### 2.3. Information sources

Information relevant to the current systematic review was searched for and identified by two investigators, Abdullah Mohammed (AM) and Musa Ahmed (MA), who conducted separate searches in research databases (Table 2) from their inception to October 15, 2023, for studies on the seroprevalence of *T. gondii* infection in workers occupationally exposed to animals, published in English. In addition to the electronic databases and grey literature sources, the authors also searched the reference lists of relevant studies for additional eligible studies.

**Table 2.** Databases searched and number of hits.

Database	Website	No. hits
Scopus	https://www.scopus.com/	71
Web of Science	https://www.webofscience.com/wos/	42
PubMed	https://pubmed.ncbi.nlm.nih.gov/	268
Google Scholar^@^	https://scholar.google.com/	317
ResearchGate^@^	https://www.researchgate.net/	197
Semantic Scholar^@^	https://www.semanticscholar.org/	68
African Journals Online (AJOL)	https://www.ajol.info/index.php/ajol	338
Scientific Electronic Library Online (SciELO)	https://www.scielo.org/	15
World cat	https://www.worldcat.org/	108
ProQuest Dissertations and Theses	https://www.proquest.com/	56
**Total**		**1480**

**Note:** ^@^ Google Scholar, Semantic Scholar, and ResearchGate were used as supplementary sources for additional relevant studies but are not listed as primary databases.

### 2.4. Search strategy and data items

The search strategy was developed in consultation with a medical librarian and tailored to each database (Table 2). The strategy aimed to identify studies that investigated the seroprevalence of *T. gondii* infection in workers occupationally exposed to animals. It employed Boolean search terms (AND, OR, NOT) and included keywords focusing on titles and abstracts related to *T. gondii* infection (Toxoplasmosis, *Toxoplasma gondii*, *T. gondii*, and Toxoplasma), workers occupationally exposed to animals (abattoir workers, animal attendants, animal handlers, animal hunters, animal hospital workers, animal technicians, butchers, dairy farmers, fishermen, fish farm workers, food animal workers, laboratory animal workers, livestock workers, meatpacking workers, slaughterhouse workers, veterinarians, veterinary pharmacists, veterinary students, and veterinary technicians), and seroprevalence (epidemiology, frequency, prevalence, risk factors, seroprevalence, and surveillance). The search covered studies published between 1972 and 2023.

### 2.5. Study selection process and risk of bias assessment

**Figure 1.**
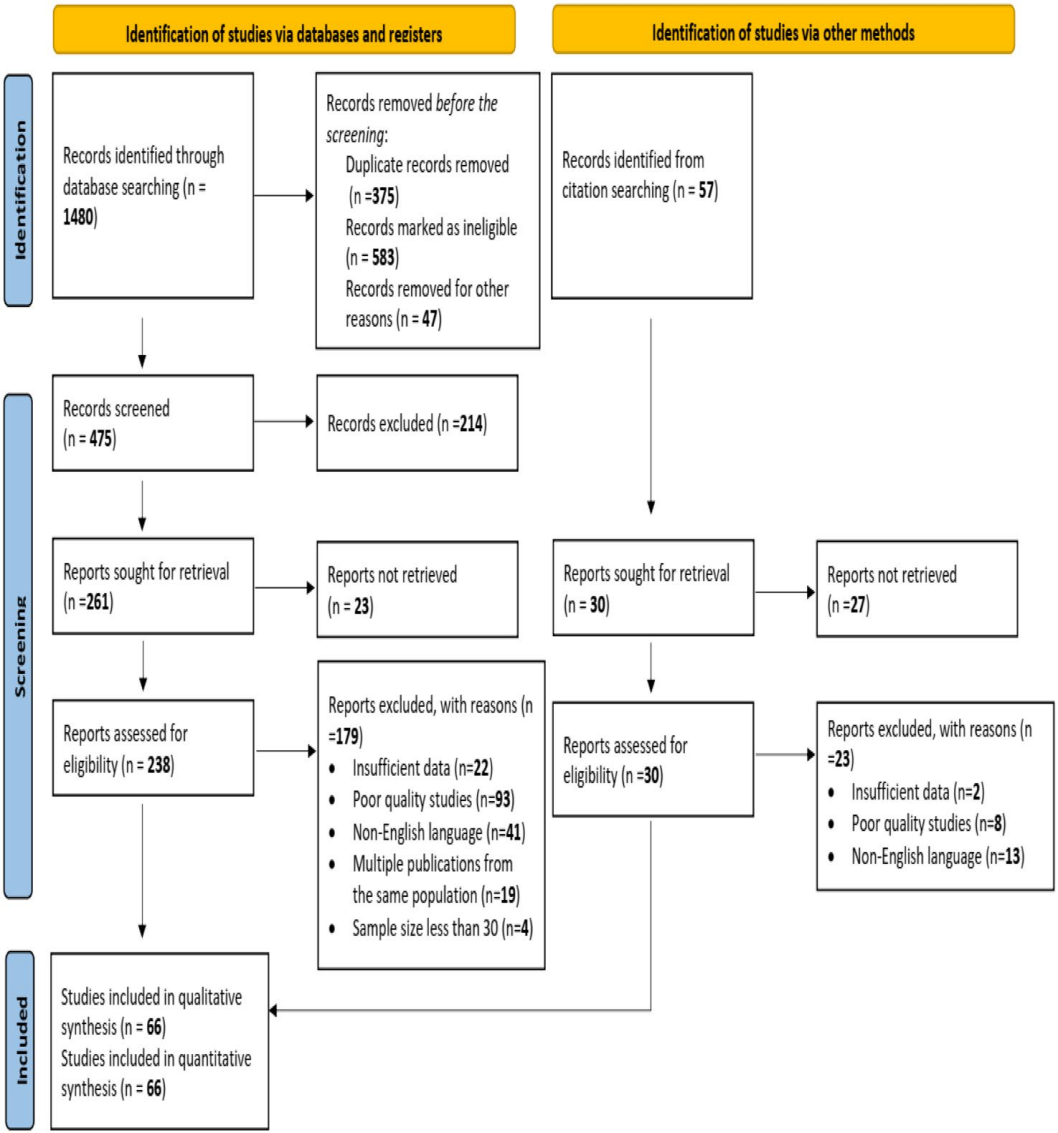
Flowchart of study inclusion and exclusion for the meta-analysis of *T. gondii* seroprevalence in WOEA.

The selection process of studies in the current analysis was guided by the PRISMA statement (Figure 1) and the abovementioned inclusion and exclusion criteria, and the following order was followed: Firstly, all selected electronic databases were screened by two independent reviewers (AM and MA) to identify and retrieve eligible studies; any disagreements during this phase between them were resolved by discussion; and the agreed studies were then exported to version X9.3.3 of the EndNote citation manager to identify and remove duplicated studies. Next, the two reviewers independently screened the titles and abstracts of all remaining studies to identify those that potentially met the inclusion criteria, and any disagreements during this phase were resolved in consultation with a third reviewer. The full text of all potentially eligible studies was then retrieved and reviewed to confirm eligibility. Subsequently, the remaining studies were subjected to additional quality checks using the Joanna Briggs Institute (JBI) critical appraisal checklist for observational studies (Jordan et al. 2019). Finally, the inter-rater agreement between AM and MA was measured using Cohen’s Kappa statistic, which achieved a value of 1, indicating perfect agreement (Gwet 2008). All studies that met the inclusion criteria of the review were selected for inclusion in the meta-analysis.

### 2.6. Data collection process

Data were collected from the full text of all eligible studies. Two independent reviewers, AM and MA, extracted data from each study using a standardized data extraction form based on the objectives of the review and the Joanna Briggs Institute data extraction tool for systematic reviews. The data extraction form included items (Table 2) related to:

- Study characteristics: author(s), study period and year of publication, study design, continent, country, and World Bank income category, which was based on the most recent World Bank income classification system that was used to categorize the participating countries based on their Gross National Income (GNI) per capita (World Bank Country and Lending Groups 2024).
- Study participant characteristics: sample size, age, gender, and occupation of participants.
- Exposure assessment: Methods used in each study to assess participant exposure to *T. gondii*.
- Outcome assessment: methods used in each study to diagnose *T. gondii* infection.
- Results: prevalence of *T. gondii* infection.

The two reviewers worked independently to extract data from each study. However, any discrepancies in the data extraction were resolved through discussion or by consultation with a third reviewer. In addition, if the reviewers had any questions about the data extracted from a study, they contacted the corresponding author of the study *via* email and/or ResearchGate personal pages to obtain or confirm the data. Finally, to ensure accuracy, a pilot test was performed on 20 randomly selected papers (30% of the total) by comparing the data extraction results of two reviewers, AM and MA. This helped identify any inconsistencies in the data extraction process and make necessary revisions to the data extraction template.

### 2.7. Synthesis methods

The results of the individual studies were synthesized and analyzed using Stata software version 16.0 and a random-effects model with a statistical significance level of *p* < 0.05.

The subsequent analysis required two statistical measurements: effect size (ES) with a 95% confidence interval (CI) and standard error (SE). Whereby, ES was calculated using the binomial distribution and the prevalence of *T. gondii* infection in workers occupationally exposed to animals (proportion), and SE was calculated using the following formula:

SE=sqrt(p(1−p)/n)

where

p is the proportion of *T. gondii* infection-positive cases in the overall population of workers who are occupationally exposed to animals.

n is the sample size.

To stabilize the variance, the Freeman-Tukey double arcsine transformation (PFT) was used before meta-analysis to make the rate more consistent with the Gaussian distribution (Mia et al. 2022; Mohammed et al. 2023).

Additionally, the presence and extent of statistical heterogeneity were assessed using the Cochran’s Q test and the I^2^ statistic. If the Cochran’s Q test was significant (*p* < 0.10) or the I^2^ statistic was greater than 50% (Higgins et al. 2003), this was considered to be evidence of statistical heterogeneity.

The present heterogeneity was explored by subgroup analyses and meta-regression to identify the possible causes of heterogeneity among study results. This involved dividing the studies in the meta-analysis into subgroups based on different characteristics, such as study design, participant characteristics, and exposure assessment, and checking the possible relationship between study variables and the prevalence of human toxoplasmosis by the meta-regression test (univariate and multivariate regression).

Furthermore, a sensitivity analysis was conducted to assess the robustness of the synthesized results by repeating the meta-analysis with different assumptions or criteria, such as excluding studies that were rated as having a ‘high’ or ‘moderate’ risk of bias.

In addition, to check for potential publication bias, the visual symmetry of the funnel plot and the result of the regression-based Egger test were used. The Egger test was considered significant if the *P* values were less than 0.10.

Overall, the results of the final meta-analysis model were presented as forest plots, which included the results of each individual study as well as the pooled results of all included studies.

## 3. Results

### 3.1. Study identification and selection

A comprehensive search strategy was employed to identify relevant studies. Electronic databases were systematically searched, yielding a total of 1480 potentially eligible studies (Table 2). Following a rigorous screening process based on pre-defined inclusion and exclusion criteria, a substantial number of studies (*n* = 1005) were excluded (Figure 1). Duplicates (*n* = 375) were removed using reference management software (EndNote version X9.3.3). An additional 583 studies were excluded for not meeting the eligibility criteria established a priori. These criteria centered on specific aspects of study design (e.g. intervention type, study population), methodological quality, and outcome measures.

The remaining 475 studies proceeded to full-text assessment for a more in-depth evaluation. Following a meticulous review process, 214 studies were excluded for various reasons. These included:

- Insufficient data for analysis (n = 22)
- Poor methodological quality as assessed by established quality appraisal tools (n = 93)
- Language barriers (studies published in languages other than English, n = 41)
- Reporting on the same study population in multiple publications (n = 19)
- Sample size less than 30 participants (n = 4)

Concurrent with the database search, a citation search of the retrieved studies identified 57 potentially relevant articles. However, 27 were excluded due to non-retrieval. The remaining 30 articles underwent full-text screening using the same stringent criteria applied to database studies. This process resulted in the exclusion of an additional 27 articles.

Ultimately, following a rigorous selection process, 66 studies were deemed eligible for inclusion in the final quantitative and qualitative synthesis. Of these, 59 originated from the initial database search, and 7 were identified through the citation search of retrieved studies.

### 3.2. Study characteristics

The key characteristics of the 66 studies included in this meta-analysis are summarized in Table 3. All continents were represented, with studies originating from Africa (*n* = 17, 25.8%), Asia (*n* = 22, 33.3%), Australia (*n* = 1, 1.5%), Europe (*n* = 9, 13.6%), North America (*n* = 9, 13.6%), and South America (*n* = 9, 13.6%). The World Bank income classifications were also well represented, with studies from high-income (*n* = 22, 33.3%), upper-middle-income (*n* = 17, 25.8%), lower-middle-income (*n* = 21, 31.8%), and low-income (*n* = 7, 10.6%) countries.

**Table 3.** Participant and main study characteristics of included studies.

Continent	Country/income classification	Study author	Period of study	Study design	Diagnostic method	Study participants occupation	Age (years)	Sample size			Seroprevalence, n (%)		
								Overall	Male	Female	Overall	Male	Female
Africa	Central African Republic/LIC	Bouscaren et al. (2018)	November 2011–December 2012.	CS	ELISA	Livestock workers	65–80	409	NR	NR	263 (64.3)	NR	NR
	Egypt/LMIC	Barakat et al. (2011)	NR	CS	IFAT	Livestock workers	NR	127	NR	NR	48 (37.8)	NR	NR
	Egypt/LMIC	Elsheikha et al. (2009)	March–September 2007	CS	ELISA	Livestock workers, slaughterhouse workers, and veterinary personnel	20–50	109	NR	NR	77 (70.6)	NR	NR
	Ghana/LMIC	Abu et al. (2015)	NR	CS	ELISA	Non-livestock workers	10–100	81	NR	NR	78 (96.3)	NR	NR
	Kenya/LMIC	Cook et al. (2021)	NR	CS	ELISA	Slaughterhouse workers	18–85	738	711	26	619 (84)	601 (97.1)	18 (2.9)
	Kenya/LMIC	Thiong’o et al. (2016)	March–June 2013	CS	PCR	Slaughterhouse workers	NR	87	NA	NA	34 (39.1)	NA	NA
	Namibia/UMIC	Colf et al. (2014)	October 2011–January 2012	CS	ELISA	Livestock workers, slaughterhouse workers, and veterinary personnel	20–50	100	NR	NR	32 (32)	NR	NR
	Nigeria/LMIC	Abraham et al. (2021)	May 2016–July 2017	CS	ELISA	Livestock workers and slaughterhouse workers	15–78	339	283	56	189 (55.8)	151 (79.9)	38 (20.1)
	Nigeria/LMIC	Alayande et al. (2012)	NR	CS	LAT	Slaughterhouse workers	11–60	75	NR	NR	20 (26.7)	NR	NR
	Nigeria/LMIC	Kamani et al. (2009)	February–May 2008	CS	ELISA	Livestock workers	NR	40	NR	NR	13 (32.5)	NR	NR
	Republic of Congo/LIC	Bouscaren et al. (2018)	November 2011–December 2012.	CS	ELISA	Livestock workers	65–80	408	NR	NR	224 (54.9)	NR	NR
	Sudan/LIC	Musa (2021)	2020–2021	CS	LAT	Livestock workers	NR	99	NR	NR	40 (40.4)	NR	NR
						Slaughterhouse workers	NR	16	NR	NR	7 (43.8)	NR	NR
						Veterinary personnel	NR	5	NR	NR	0	NR	NR
						Overall	16–68	120	66	54	47 (39.2)	32 (68.1)	15 (31.9)
	Sudan/LIC	Idris (2021)	February 2020–July 2021	CS	LAT	Livestock workers	NR	77	NR	NR	37 (48.1)	NR	NR
						Slaughterhouse workers	NR	29	NR	NR	12 (41.4)	NR	NR
						Veterinary personnel	NR	14	NR	NR	5 (35.7)	NR	NR
						Overall	6–80	120	78	42	54 (45)	38 (70.4)	16 (29.6)
	Sudan/LIC	Mohamed (2021)	February 2020–July 2021	CS	LAT	Livestock workers	NR	86	NR	NR	29 (33.7)	NR	NR
						Slaughterhouse workers	NR	21	NR	NR	13 (61.9)	NR	NR
						Veterinary personnel	NR	13	NR	NR	9 (69.2)	NR	NR
						Overall	1–100	120	56	64	51 (42.5)	27 (52.9)	24 (47.1)
	Sudan/LIC	Ahmed (2021)	February 2020–September 2021	CS	LAT	Livestock workers	NR	105	NR	NR	84 (80)	NR	NR
						Slaughterhouse workers	NR	6	NR	NR	4 (66.7)	NR	NR
						Veterinary personnel	NR	9	NR	NR	8 (88.9)	NR	NR
						Overall	1–80	120	30	90	96 (80)	27 (28.1)	69 (71.9)
	Sudan/LIC	Alshaeb (2017)	November 2017	CC	LAT	Slaughterhouse workers & veterinarians	NR	70	NA	NA	8 (11.4)	NA	NA
	Tanzania/LMIC	Swai and Schoonman (2009)	November 2005	CS	LAT	Livestock workers	NR	67	NR	NR	35 (52.2)	NR	NR
						Slaughterhouse workers	NR	41	NR	NR	19 (46.3)	NR	NR
						Non-livestock workers	NR	38	NR	NR	15 (39.5)	NR	NR
						Veterinary personnel	NR	11	NR	NR	4 (36.4)	NR	NR
						Overall	14–84	199	132	67	91 (45.7)	62 (68.1)	29 (31.9)
Asia	China/UMIC	Mao et al. (2021)	2020	CS	ELISA	Livestock workers, and slaughterhouse workers	16–92	1330	606	724	154 (11.6)	76 (49.4)	78 (50.6)
	India/LMIC	Deshmukh et al. (2021)	2020	CS	ELISA	Veterinary personnel	NR	139	NR	NR	68 (48.9)	NR	NR
						Slaughterhouse workers^@^	25–45	126	NR	NR	61 (48.4)	NR	NR
						Overall	NR	265	NR	NR	129 (48.7)	NR	NR
	India/LMIC	Thakur et al. (2022)	2017–18	CS	ELISA	Veterinary personnel	21–60	205	NR	NR	18 (9)	NR	NR
	India/LMIC	Rahman et al. (2008)	2004–2005	CS	ELISA	Veterinary personnel	NR	78	NR	NR	8 (10.3)	NR	NR
	Iraq/UMIC	Al-Imara and Thamir (2009)	NR	CS	LAT	Slaughterhouse workers	20–50	100	NR	NR	47 (47)	NR	NR
	Iraq/UMIC	Omer (2008)	2004–2005	CS	LAT	Slaughterhouse workers	20–50	79	NR	NR	44 (55.7)	NR	NR
	Iran/LMIC	Hejazi et al. (2023)	2021	CC	ELISA	Livestock workers, slaughterhouse workers, and veterinary personnel	NR	401	NR	NR	185 (46.1)	NR	NR
	Iran/LMIC	Beheshtipour et al. (2019)	May–October 2018	CS	ELISA	Slaughterhouse workers	NR	53	NR	NR	6 (11.3)	NR	NR
	Iran/LMIC	Youssefi et al. (2018)	2016	CS	ELISA	Slaughterhouse workers	NR	91	NR	NR	53 (58.2)	NR	NR
	Iran/LMIC	Rostami et al. (2016)	July 2014–March 2015	CS	ELISA	Livestock workers	NR	57	NR	NR	46 (80.7)	NR	NR
	Iran/LMIC	Mardani and Tavalla (2015)	2014	CC	ELISA	Slaughterhouse workers	NR	110	NR	NR	56 (50.9)	NR	NR
	Iran/LMIC	Sadaghian and Jafari (2014)	NR	CC	ELISA	Veterinary personnel	NR	80	NR	NR	27 (33.8)	NR	NR
	Iran/LMIC	Shad-Del et al. (1993)	NR	CS	IFAT	Livestock workers	NR	50	NR	NR	14 (28)	NR	NR
						Slaughterhouse workers	NR	50	NR	NR	17 (34)	NR	NR
						Veterinary personnel	NR	50	NR	NR	5 (10)	NR	NR
						Overall	18–67	150	NR	NR	36 (24)	NR	NR
	Japan/HIC	Horio et al. (2001)	1992–1993	CS	LAT	Slaughterhouse workers	20–60	67	32	35	22 (32.8)	8 (36.4)	14 (63.6)
	Malaysia/UMIC	Brandon-Mong et al. (2015)	October 2013–April 2014	CS	ELISA	Veterinary personnel	17–64	312	78	234	62 (19.9)	23 (37.1)	39 (62.9)
	Malaysia/UMIC	Normaznah et al. (2004)	NR	CS	IFAT	Livestock workers	NR	79	NR	NR	22 (27.8)	NR	NR
	Myanmar/LMIC	Sint et al. (2023)	June–December 2020	CS	RDT	Slaughterhouse workers	18–66	139	119	20	61 (43.9)	53 (86.9)	8 (13.1)
	Pakistan/LMIC	Khan et al. (2022)	NR	CS	ICT	Slaughterhouse workers	15–31	270	NR	NR	59 (21.9)	NR	NR
	Pakistan/LMIC	Anees et al. (2014)	NR	CS	LAT	Slaughterhouse workers	51–60	50	NR	NR	5 (10)	NR	NR
	Republic of Korea/HIC	Sang-Eun et al. (2014)	2009	CS	EIA	Veterinary personnel	30–60	945	NR	NR	76 (8)	NR	NR
	Saudi Arabia/HIC	Mohamed et al. (2020)	March–April 2019	CS	ELISA	Slaughterhouse workers	22–61	108	NA	NA	27 (25)	NA	NA
	Saudi Arabia/HIC	Amin and Morsy (1997)	NR	CS	ELISA	Slaughterhouse workers	NR	100	NR	NR	80 (80)	NR	NR
	Taiwan/HIC	Fan et al. (2001)	July 1999–June 2000	CS	LAT	Non-livestock workers	14–60	53	NR	NR	28 (52.8)	NR	NR
Australia	New Zealand/HIC	Forsyth et al. (2012)	2002–2010	CS	ELISA	Veterinary personnel & Non-livestock workers	NR	88	NR	NR	38 (43.2)	NR	NR
Europe	Denmark/HIC	Lings et al. (1994)	September 1986	CS	ELISA	Slaughterhouse workers	16–66	217	203	14	98 (45.2)	NR	NR
	Estonia/HIC	Lassen et al. (2016)	March 2013–January 2014	CS	ELISA	Livestock workers	NR	375	NR	NR	281 (74.7)	NR	NR
			October 2012			Veterinary personnel	NR	158	NR	NR	73 (46.2)	NR	NR
			July 2013			Non-livestock workers	NR	144	NR	NR	94 (65.3)	NR	NR
			October 2012–January 2014			Overall	NR	677	NR	NR	451 (66.6)	NR	NR
	Finland/HIC	Seuri and Koskela (1992)	1989	CS	ELISA	Livestock workers	20–65	53	41	12	20 (37.7)	15 (75)	5 (25)
						Slaughterhouse workers	20–65	40	36	4	10 (25)	9 (90)	1 (10)
						Overall	20–65	93	77	16	30 (32.3)	24 (80)	6 (20)
	Northern Ireland/HIC	Stanford et al. (1990)	NR	CS	IHAT	Livestock workers	18–83	407	NR	NR	299 (73 5)	NR	NR
	Slovak Republic/HIC	Fecková et al. (2020)	NR	CS	ELISA	Livestock workers	NR	219	NR	NR	93 (42.5)	NR	NR
						Veterinary personnel	NR	294	NR	NR	40 (13.6)	NR	NR
						Non-livestock workers	NR	196	NR	NR	54 (27.6)	NR	NR
						Overall	NR	709	NR	NR	187 (26.4)	NR	NR
	Türkiye/UMIC	Acici et al. (2008)	NR	CS	ELISA	Livestock workers	NR	72	32	40	23 (31.9)	8 (34.8)	15 (65.2)
	Poland/HIC	Wójcik-Fatla et al. (2018)	June–December 2017	CS	ELFA	Veterinary personnel	30–61	373	162	211	166 (44.5)	90 (54.2)	76 (45.8)
	Portugal/HIC	Almeida et al. (2022b)	NR	CC	ELISA	Veterinary personnel	19–63	350	98	252	91 (26)	25 (27.5)	66 (72.5)
	Portugal/HIC	Almeida et al. (2022a)	NR	CS	ELISA	Livestock workers, slaughterhouse workers, and veterinary personnel	20–83	114	79	35	83 (72.8)	59 (71.1)	24 (28.9)
North America	Canada/HIC	Shuhaiber et al. (2003)	2002	CS	ELISA	Veterinary personnel	30–45	141	NR	NR	20 (14.2)	NR	NR
	Mexico/UMIC	Alvarado-Esquivel et al. (2014)	August 2013–July 2014	CC	EIA	Livestock workers	18–67	61	NR	NR	2 (3.3)	NR	NR
						Veterinary personnel		139	NR	NR	10 (7.2)	NR	NR
						Overall		200	NR	NR	12 (6)	NR	NR
	Mexico/UMIC	Alvarado-Esquivel et al. (2011)	September 2009–October 2010	CC	EIA	Slaughterhouse workers	16–71	124	103	21	7 (5.7)	6 (85.7)	1 (14.3)
	Mexico/UMIC	Galván-Ramírez et al. (2008)	NR	CS	ELISA	Slaughterhouse workers	NR	145	NR	NR	104 (71.7)	NR	NR
	USA/HIC	Rosypal et al. (2015)	2002–2006	CS	ELISA	Veterinary personnel	NR	336	68	268	16 (4.8)	11 (68.7)	5 (31.3)
	USA/HIC	Weigel et al. (1999)	1993	CS	MAT	Livestock workers	18–83	174	NR	NR	54 (31)	NR	NR
	USA/HIC	DiGiacomo et al. (1990)	September 1979–March 1981	CS	IFAT	Veterinary personnel	NR	61	NR	NR	23 (37.7)	NR	NR
	USA/HIC	Sengbusch and Sengbusch (1976)	NR	CC	IFAT	Veterinary personnel	15–66	60	NR	NR	11 (18.3)	NR	NR
	USA/HIC	Riemann et al. (1974)	NR	CS	IFAT	Veterinary personnel	NR	138	86	52	27 (19.6)	15 (55.6)	12 (44.4)
South America	Brazil/UMIC	Clazer et al. (2017)	May–November 2014	CS	IFAT	Veterinary personnel	NR	157	83	74	46 (29.3)	20 (43.5)	26 (56.5)
	Brazil/UMIC	Vicente et al. (2014)	NR	CC	ELISA/IFAT	Veterinary personnel	NR	839	NR	NR	183 (21.8)	NR	NR
	Brazil/UMIC	Gonçalves et al. (2006)	July–September 2003	CS	IFAT	Slaughterhouse workers	NR	150	113	37	105 (70)	80 (76.2)	25 (23.8)
	Brazil/UMIC	Dias et al. (2005)	December 2002–January 2003	CS	IFAT	Slaughterhouse workers	NR	47	43	4	28 (59.6)	25 (89.3)	3 (10.7)
	Brazil/UMIC	Riemann et al. (1975)	January–July 1972	CS	IFAT	Slaughterhouse workers	NR	144	124	20	103 (71.5)	86 (83.5)	17 (16.5)
	Brazil/UMIC	Riemann et al. (1974)	NR	CS	IFAT	Veterinary personnel	NR	219	26	193	100 (45.7)	12 (12)	88 (88)
	Chile/HIC	Toro et al. (2017)	NR	CS	CLIA	Slaughterhouse workers, and veterinary personnel	NR	39	NR	NR	24 (61.5)	NR	NR
	Colombia/UMIC	Molina-Guzmán et al. (2019)	NR	CS	ELISA	Livestock workers	19–76	328	286	42	156 (47.6)	136 (87.2)	20 (12.8)
	Trinidad and Tobago/HIC	Adesiyun et al. (2011)	NR	CS	EIA	Livestock workers	18–60	394	313	81	150 (38.1)	119 (79.3)	31 (20.7)
						Slaughterhouse workers		99	92	7	44 (44.4)	39 (88.6)	5 (11.4)
						Overall		493	405	88	194 (39.4)	158 (81.4)	36 (18.6)

**Abbreviations:** *CC*; Case-control study, *CS*; Cross-sectional study, CLIA; Chemiluminescence immunoassay, *EIA*; Enzyme immunoassay, *ELISA*; enzyme-linked immunosorbent assay, *ELFA*; Enzyme-linked fluorescence assays, *HIC*, high-income countries; *ICT*; Immuochromatography test, *IFAT*; Indirect fluorescent antibody technique, *IHAT*; modified agglutination test, *LAT;* latex agglutination test, *LIC*; low income countries, *LMIC*; low middle-income countries, *NR*; Not reported, *MAT*; modified agglutination test, *RDT*; Rapid diagnostic testing, *UMIC*; upper middle-income countries. **Note**: @Slaughterhouse workers from India were only involved in the slaughtering and selling of livestock (sheep and goat) and poultry meat; veterinary personnel (Professor/academicians, veterinary doctors, veterinary students working in clinics, veterinary pharmacists, veterinary technicians, and animal hospital workers).

Slaughterhouse workers were the most frequently studied population (*n* = 35, 53.0%), followed by veterinary personnel and livestock workers (*n* = 25 each, 37.9%). Only 9.1% of studies (*n* = 6) explored prevalence in non-livestock workers (animal attendants, handlers, hunters, fishermen, fish farm workers, and laboratory animal workers). Publication dates ranged from before 2000 (*n* = 13, 19.7%) to after 2010 (*n* = 37, 56.1%), with the most published between 2000 and 2010 (*n* = 17, 25.8%). All studies employed observational designs, with the vast majority being cross-sectional (*n* = 56, 84.8%) and a smaller number being case-control (*n* = 10, 15.2%).

A variety of diagnostic methods were used. Enzyme-linked immunosorbent assays (ELISA) were the most common (*n* = 32, 48.5%), followed by latex agglutination tests (LAT) (*n* = 12, 18.2%), indirect fluorescent antibody technique (IFAT) (*n* = 11, 16.7%), and enzyme immunoassay (EIA) (*n* = 4, 6.1%). Less frequent methods included enzyme-linked fluorescence assays (ELFA), indirect hemagglutination test (IHAT), modified agglutination test (MAT), chemiluminescence immunoassays (CLIA), immunochromatographic tests (ICT), rapid diagnostic testing (RDT), and polymerase chain reaction (PCR) (all *n* = 1, 1.5% each).

It is important to note that only 26 of the included studies (39.4%) reported data on the effect of gender on the prevalence of *T. gondii* infection.

### 3.3. Results of synthesis

**Figure 2.**
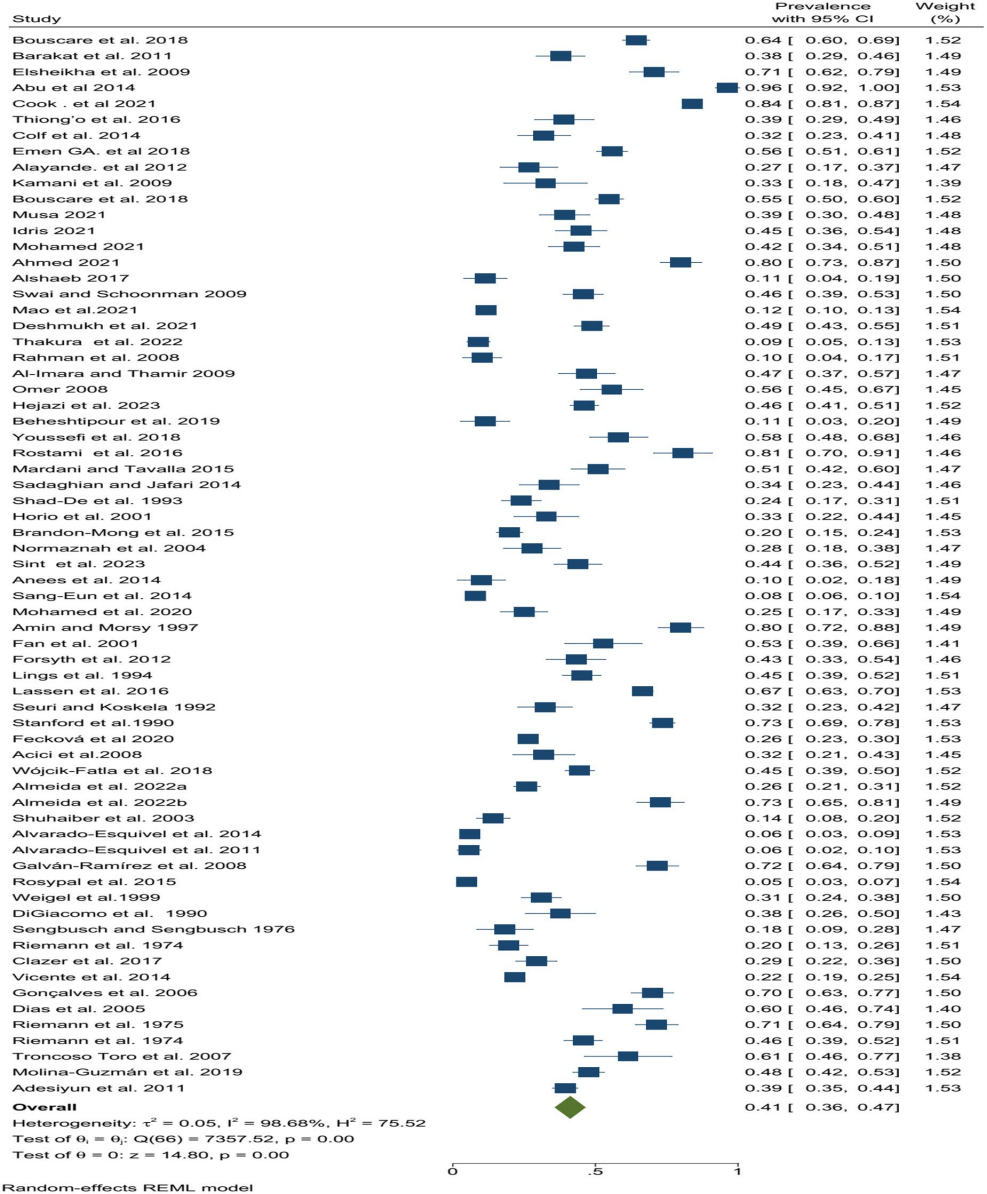
Forest plot (random-effects model) of pooled *T. gondii* seroprevalence in WOEA worldwide.

This meta-analysis included data from 66 studies encompassing a total of 15,279 participants occupationally exposed to animals. The prevalence of *T. gondii* infection exhibited significant heterogeneity across the studies (*p* < 0.0001; I^2^ = 98.68%; τ^2^ = 0.05). This high level of heterogeneity indicates substantial variation in prevalence estimates between studies. Due to this heterogeneity, a random-effects model (REML) was employed for the final analysis. The pooled prevalence of *T. gondi*i infection in workers occupationally exposed to animals across the globe was estimated to be 41% (95% CI: 36–47%) (Figure 2). This finding suggests a substantial burden of this parasitic infection among this occupational group. However, it is crucial to acknowledge the wide range of prevalence estimates reported in individual studies, ranging from a low of 4.8% (North America, USA, 2002–2006) to a high of 96.3% (Africa, Ghana, 2010s) (Table 4). This variation highlights the potential influence of factors such as geographic location, animal contact patterns, and diagnostic methods employed in the studies.

**Table 4.** Pooled prevalence estimates and heterogeneity measures of *T. gondii* infection in WOEA by subgroup characteristics.

Analysis of *T. gondii* infection		Pooled estimates				Heterogeneity				
		No of studies	Pooled sample size	Cases	Pooled prevalence % (95% CI)	Q-value	tau2	I^2^%	H^2^	Q-*p* value
Gender	Male	26	2621	554	63 (54–72)	1486.96	0.051	97.5	40.07	**<0.001**
	Female	26	2621	481	37 (28–46)	1486.96	0.051	97.5	40.07	**<0.001**
Study participants occupation	Livestock workers	25	4776	2263	47 (38–55)	1693.45	0.042	97.71	43.67	**<0.001**
	Non-livestock workers	6	600	307	54 (35–74)	381.64	0.059	97.4	38.52	**<0.001**
	Slaughterhouse workers	35	4728	2187	44 (37–51)	2469.81	0.042	97.09	34.41	**<0.001**
	Veterinary personnel	25	5126	1096	27 (19–34)	1620.38	0.036	99.23	129.7	**<0.001**
Continent	Africa	17	3262	1944	51 (40–62)	963.85	0.049	98.04	51.13	**<0.001**
	Asia	22	5122	1251	36 (26–45)	1100.96	0.048	98.65	74.23	**<0.001**
	Australia	1	88	38	43 (33–54)	0.00	0.000	NE	NE	**<0.001**
	Europe	9	3012	1428	47 (34–60)	555.02	0.038	98.25	57.05	**<0.001**
	North America	9	1379	274	23 (9–37)	367.6	0.045	98.66	74.43	**<0.001**
	South America	9	2416	939	49 (38–61)	322.55	0.030	97.08	34.28	**<0.001**
World Bank country income classification	High-income	22	5743	2045	39 (30–48)	2225.28	0.046	98.73	78.99	**<0.001**
	Upper-middle income	17	4425	1228	38 (28–49)	1042.54	0.049	98.76	80.56	**<0.001**
	Lower-middle income	21	3744	1858	44 (33–54)	1987.17	0.061	98.36	61.06	**<0.001**
	Low-income	7	1367	743	48 (32–64)	216.03	0.045	97.49	39.87	**<0.001**
Study year/s	1972–1981	4	561	241	39 (14–64)	128.31	0.062	97.74	44.25	**<0.001**
	1982–1991	2	468	322	56 (21–91)	29.61	0.062	96.62	29.61	**<0.001**
	1992–2001	7	854	348	43 (28–57)	136.61	0.035	95.21	20.89	**<0.001**
	2002–2011	19	3391	985	39 (28–49)	1198.87	0.051	98.6	71.21	**<0.001**
	2012–2023	35	10005	3978	42 (34–50)	4626.56	0.055v	98.93	93.28	**<0.001**
Overall		**66**	**15279**	**5874**	**41 (36**–**47)**	**7357.52**	**0.05**	**98.68**	**75.52**	**<0.001**

### 3.4. Subgroup analysis

To address the substantial heterogeneity observed in the overall pooled prevalence estimate (I^2^ = 98.68%), a subgroup analysis was conducted to explore the potential influence of various factors on *T. gondii* infection rates among workers with occupational animal exposure (presented in Table 4).

#### 3.4.1. Gender

**Figure 3.**
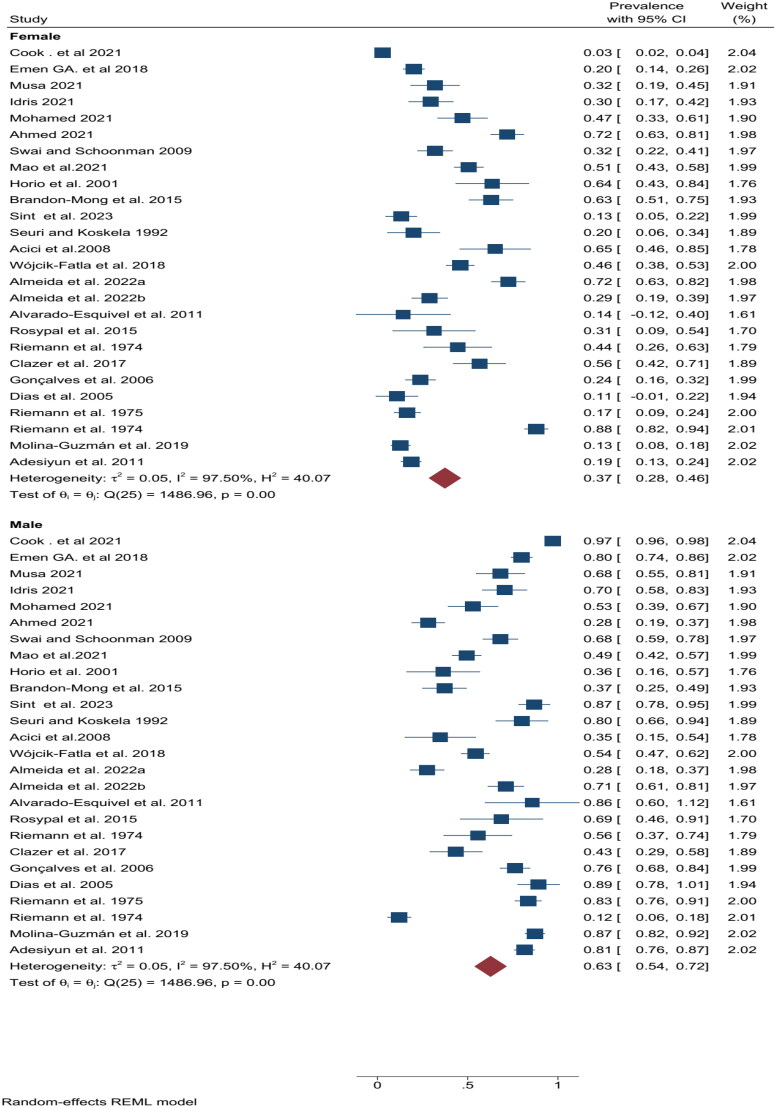
Forest plot (random-effects model) of pooled *T. gondii* seroprevalence in WOEA worldwide, stratified by gender.

The analysis revealed statistically significant differences in prevalence by gender (*p* < 0.05). Males exhibited a higher pooled prevalence (63%) compared to females (37%) (Figure 3). This translates to a prevalence ratio (PR) of approximately 1.7. In other words, males in this study were about 1.7 times more likely to have the condition than females.

#### 3.4.2. Occupation

**Figure 4.**
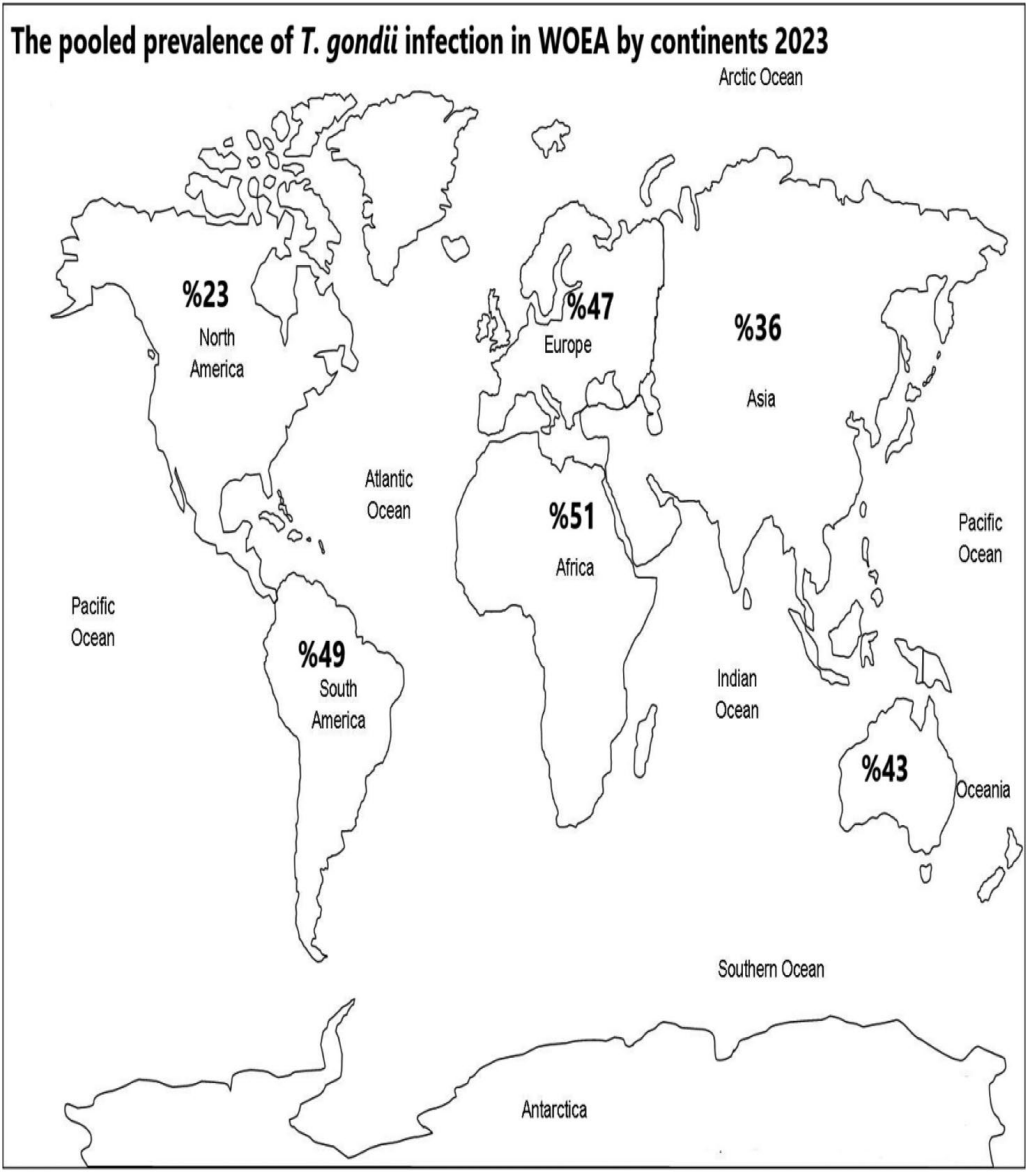
The pooled seroprevalence of *T. gondii* infection in WOEA by continents.

Occupation also emerged as a significant factor influencing prevalence (*p* < 0.05). Non-livestock workers displayed the highest prevalence (54%), followed by livestock workers (47%), slaughterhouse workers (44%), and veterinary personnel (27%)(Figure 4).

#### 3.4.3. Geographic region

A clear geographic trend was observed in the prevalence of *T. gondii* infection. Workers in Africa exhibited the highest prevalence (51%), followed by South America (49%), Europe (47%), Australia (43%), Asia (36%), and North America (23%).

#### 3.4.4. World bank income classification

Interestingly, the analysis revealed a trend of lower prevalence in high-income (39%) and upper-­middle-income (38%) countries compared to lower-­middle-income (44%) and low-income (48%) countries. While statistically significant (*p* < 0.05), further investigation is warranted to elucidate the underlying mechanisms behind this association.

#### 3.4.5. Study period

The subgroup analysis indicated a slight fluctuation in prevalence based on the study period, but no statistically significant temporal trend was evident (*p* > 0.05).

#### 3.4.6. Diagnostic method

The prevalence of *T. gondii* infection was reported separately based on the type of laboratory test used. The studies employed both molecular tests (e.g. PCR) and serological tests (e.g. ELISA and IFAT). The pooled prevalence for studies using serological tests was 41% (95% CI: 36–47%), while the pooled prevalence for studies using molecular tests was 39% (95% CI: 28–49%).

Overall, this subgroup analysis provided valuable insights into potential sources of heterogeneity in the prevalence of *T. gondii* infection among workers with occupational animal exposure. These findings highlight the importance of considering gender, occupation, geographic location, and socioeconomic status when evaluating the risk of *T. gondii* infection in this population group.

### 3.5. Meta-regression, sensitivity analysis, and publication bias

**Figure 5.**
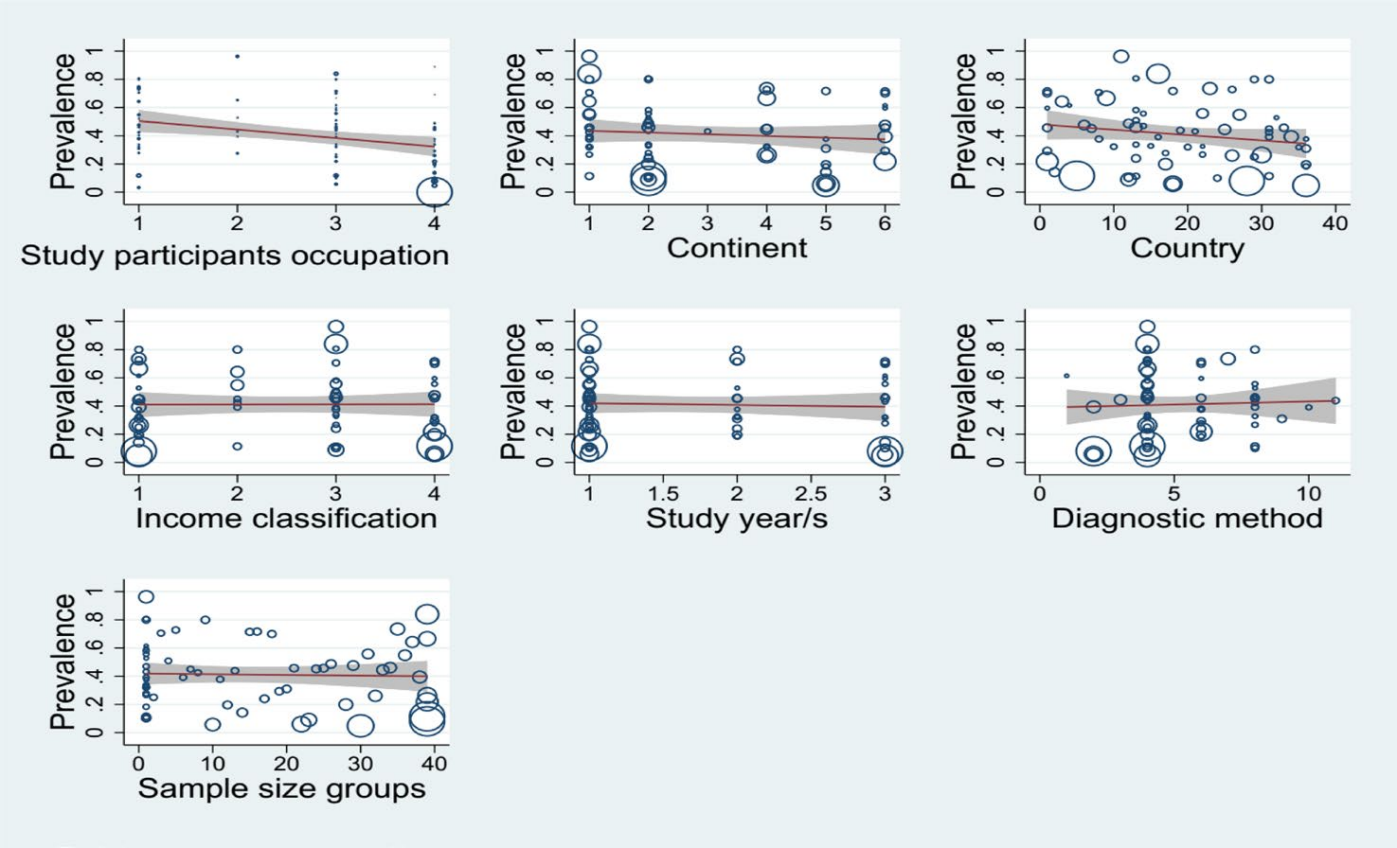
Meta-regression analysis: scatter plot depicting the relationship between study variables and pooled *T. gondii* seroprevalence estimates in WOEA.

To further explore the sources of heterogeneity in the overall prevalence estimate, a meta-regression analysis was conducted. This analysis investigated the potential association between various study characteristics and the prevalence of *T. gondii* infection (Figure 5). All examined variables, except study design, displayed a statistically significant association with prevalence in the univariate meta-regression analysis. However, when a multivariate meta-regression model was employed, only the country of the study participants remained statistically significant (Table 5). This suggests that country-specific factors likely account for a substantial portion of the observed heterogeneity between studies.

**Table 5.** Random-effects meta-regression analysis of factors associated with *T. gondii* seroprevalence in WOEA.

Univariate regression							
Variables	Coefficient	SE	Z	*p* Value	95%CI	I^2^	R^2^
Study participants occupation	0.566	0.057	9.96	<0.001	(0.454–0.677)	98.71	9.87%
Continent	0.448	0.054	8.30	<0.001	(0.342–0.554)	98.65	0.001%
Country	0.481	0.054	8.86	<0.001	(0.375 − 0.588)	98.59	1.78%
Income classification	0.41	0.065	6.29	<0.001	(0.282–0.537)	98.63	0.001%
Study period	0.433	0.063	6.9	<0.001	(0.31–0.556)	98.62	0.001%
Study design	0.091	0.142	0.64	0.523	(-0.188–0.369)	98.56	6.29%
Diagnostic method	0.389	0.076	5.12	<0.001	(0.24–0.537)	98.66	0.001%
Sample size	0.419	0.041	10.35	<0.001	(0.34–0.498)	98.67	0.001%
Multivariate regression							
Variables	Coefficient	SE	Z	*p* Value	95%CI		
Intercept	0.63	0.165	3.82	<0.001	(0.307–0.954)	98.46	0.001%
Continent	−0.017	0.018	−0.96	0.338	(-0.052–0.0177)		
Country	−0.006	0.003	−2.00	0.045	(-0.013–0.0001)		
Income classification	−0.031	0.028	−1.10	0.273	(-0.086–0.024)		
Study period	−0.005	0.037	−0.12	0.902	(-0.077–0.068)		
Diagnostic method	0.007	0.015	0.51	0.611	(-0.022–0.037)		
Sample size	−0.001	0.002	−0.17	0.864	(-0.005–0.004)		

A sensitivity analysis was performed to assess the robustness of the meta-analysis findings. This analysis involved sequentially excluding studies from the model and evaluating any resulting changes in the pooled prevalence estimate. No significant differences in the pooled prevalence were observed, indicating that the overall findings were relatively stable and not unduly influenced by any single study (see details).

**Figure 6.**
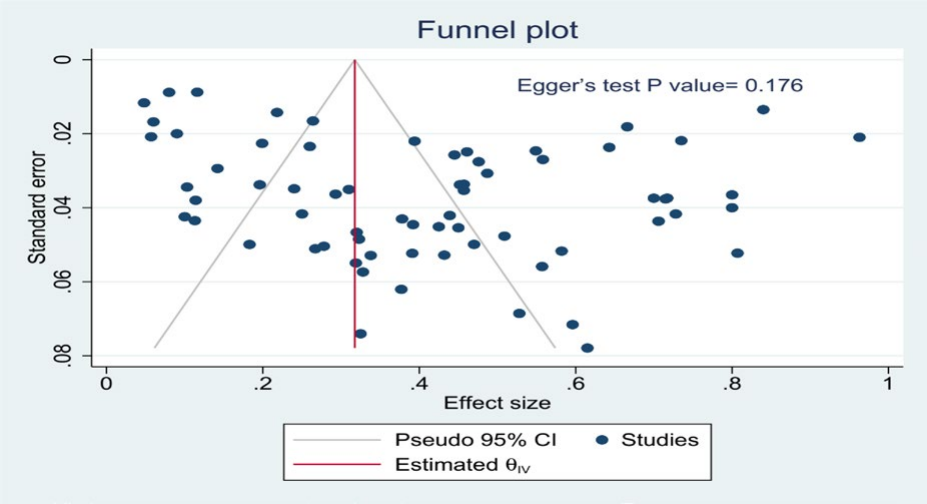
Funnel plot for evaluation of publication bias in the meta-analysis of pooled *T. gondii* seroprevalence in WOEA worldwide.

Finally, potential publication bias was evaluated using two methods: a funnel plot (Figure 6) and Egger’s regression test. The funnel plot displayed a symmetrical appearance, and Egger’s test did not yield a statistically significant result (*p* = 0.176). These findings suggest a low likelihood of publication bias significantly influencing the meta-analysis results.

## 4. Discussion

This systematic review and meta-analysis underscore the significant public health burden of *T. gondii* infection among workers with occupational animal exposure (WOEA) on a global scale. The high pooled prevalence of 41% (95% CI: 36–47%), derived from 66 studies encompassing over 15,000 participants, translates to a significant proportion of WOEA workers testing positive for antibodies against *T. gondii*, indicating a past or current infection.

The significance of this elevated prevalence lies in the clear distinction between WOEA workers and the general population. While existing research suggests that around 30% of the general population carries *T. gondii* antibodies (Rahmanian et al. 2020), our analysis demonstrates a demonstrably higher rate of infection, specifically within the WOEA group.

This disparity underscores the heightened risk faced by WOEA workers due to their occupational exposure to animals. The nature of their jobs likely increases their chances of encountering *T. gondii*, a parasite commonly found in animals like cats and some livestock.

The observed heterogeneity in prevalence across studies (I^2^ = 98.68%) suggests the influence of various factors. Notably, our analysis revealed a significant geographic disparity in seroprevalence, with the highest burden observed in African WOEA (51%). This finding aligns with previous research on zoonotic diseases like Brucella and tuberculosis in WOEA, highlighting the increased risk of occupational zoonoses in Africa compared to other regions (Mia et al. 2022).

Several factors are likely to contribute to this geographic disparity. Hot and humid climates prevalent in many African regions favor the sporulation and environmental persistence of *T. gondii* oocysts (Afonso et al. 2013; Yan et al. 2016). This extended environmental survival period translates to a heightened exposure risk for WOEA engaged in outdoor work and having frequent contact with soil or potentially contaminated areas.

Furthermore, animal husbandry practices in Africa, such as free-roaming livestock, can contribute to widespread oocyst contamination of pastures and grazing areas, thereby increasing exposure risk for WOEA herding or tending to these animals (Seo and Mendelsohn 2008). Limited access to veterinary care and preventive measures for animal diseases in some African countries further exacerbates the situation. This, coupled with the potential impact of wars and armed conflicts on the continent (Mohammed and Ahmed 2023), might contribute to a higher prevalence of *T. gondii* in animal populations, ultimately increasing the risk of transmission to WOEA.

Lower socioeconomic development in some African countries can also play a role. Poorer sanitation practices and limited access to clean water can increase environmental exposure to oocysts for both WOEA and the general population. Additionally, cultural practices involving the consumption of raw or undercooked meat, more common in some parts of Africa (Abdelnabi et al. 2016), pose a higher risk for WOEA who handle or slaughter animals.

The observed geographic disparity suggests a potential research gap in *T. gondii* infection among WOEA in certain African regions compared to developed countries.

This gap could lead to an underestimation of the true prevalence and hinder the development of targeted preventive measures. To address this disparity and protect at-risk populations, we propose increased awareness campaigns among WOEA and healthcare professionals regarding *T. gondii* infection and its transmission routes. Improved knowledge can empower WOEA to adopt preventive behaviors and facilitate early diagnosis.

The trend of lower seroprevalence in high-income countries suggests a potential influence of socioeconomic factors on *T. gondii* exposure among WOEA. Improved sanitation practices, access to clean water, and better veterinary care for animals, all more prevalent in developed nations, can significantly reduce environmental oocyst contamination.

The current analysis revealed an intriguing trend within the WOEA population. Non-livestock workers exhibited the highest pooled prevalence of *T. gondii* infection (54%), followed by livestock workers and slaughterhouse workers. Interestingly, veterinary personnel displayed the lowest prevalence (27%). Several potential explanations warrant further investigation.

One possibility relates to exposure dynamics. The broad category of ‘non-livestock workers’ likely encompasses a diverse range of professions with varying degrees and types of animal contact. This includes zookeepers, wildlife rehabilitators, shearers, fishermen, fish farm workers, animal hunters, or personnel in pet stores and grooming facilities. These jobs might involve exposure to a wider variety of animal species compared to those working primarily with livestock. A broader host range of exposure could increase the chance of encountering infected animals, potentially explaining the higher prevalence observed in non-livestock workers.

Conversely, livestock and slaughterhouse workers, while still at risk, might develop a degree of immunological tolerance through repeated low-dose exposures to *T. gondii* within specific livestock populations (e.g. cattle, pigs, and sheep) (Sana et al. 2022).

Another potential explanation is the influence of hygiene practices and biosecurity protocols. Veterinary personnel are likely to be more cognizant of the risks associated with *T. gondii* and implement stricter hygiene protocols in their work environment. This could encompass consistent use of personal protective equipment (PPE) like gloves and masks, adherence to meticulous handwashing procedures, and maintaining a sanitized workspace (Odo et al. 2015; Habib and Alshehhi 2021; Kimindu et al. 2024). These practices can significantly mitigate the risk of oocyst ingestion.

In contrast, adherence to hygiene protocols might be less consistent among non-livestock workers and some livestock workers, depending on the specific job role and workplace culture. Jobs with a higher risk of exposure, such as shearing or handling birthing animals, might necessitate more stringent hygiene practices compared to roles with less frequent direct animal contact. Additionally, the availability and accessibility of PPE and handwashing facilities within the workplace can influence adherence to hygiene protocols.

Further research is needed to elucidate the specific reasons behind these observed variations. Investigating the specific work activities and animal contact patterns within each WOEA category could provide valuable insights into exposure dynamics. Additionally, exploring the level of training and awareness about *T. gondii* infection and preventive measures across different professions could shed light on potential discrepancies in hygiene practices and risk perception. By examining these factors in a comprehensive manner, researchers and public health professionals can develop more targeted interventions to effectively reduce the risk of *T. gondii* infection for all WOEA populations.

Our subgroup analysis revealed a statistically significant difference in *T. gondii* infection prevalence by gender (*p* < 0.05), with males exhibiting a higher pooled prevalence compared to females. This disparity may be attributable to a confluence of factors, including potential behavioral and occupational variations. Certain occupations within the WOEA category might be more male-dominated and involve activities associated with a higher risk of *T. gondii* exposure. Examples include jobs in shearing, herding, or cull animal disposal, which often fall under the non-livestock worker category. These roles may involve more frequent and direct contact with bodily fluids or contaminated environments compared to positions typically filled by females (Habib et al. 2014). Additionally, gender-based differences in risk perception and adherence to hygiene protocols could contribute to the observed disparity. Males might be less likely to perceive the importance of meticulous handwashing or the consistent use of personal protective equipment (PPE), potentially increasing their risk of accidental oocyst ingestion.

This review’s findings yielded significant insights for policymakers and healthcare professionals regarding the historical burden of *T. gondii* infection within the WOEA group. This information informed the development and implementation of interventions to mitigate infection risk and enhance toxoplasmosis management in this population group.

Policymakers may leverage the review’s findings to formulate evidence-based policies aimed at curtailing *T. gondii* infection risk within the WOEA population group. This could entail the development of regulations mandating employers furnish workers with suitable PPE and training. Additionally, the establishment of funding programs to support employers in implementing these measures might be undertaken.

Healthcare professionals may utilize the review’s insights to refine the management of toxoplasmosis within the WOEA population. This could involve the development of screening programs to identify infected workers and provide them with appropriate treatment and counseling. Furthermore, the creation of educational materials to educate workers on *T. gondii* infection risks and preventative measures might have been implemented.

Public health authorities may develop targeted educational campaigns during the relevant timeframe to raise awareness concerning *T. gondii* infection risks and the significance of preventative measures. Studying the historical prevalence of *T. gondii* infection within the WOEA population served the dual purpose of safeguarding worker health and promoting broader public health initiatives, as *T. gondii* possesses the capacity for transmission from pregnant women to their fetuses and through foodborne illness. By comprehending the historical prevalence of infection among workers handling food animals, public health officials are better equipped to identify and mitigate potential public health risks.

The current study acknowledges limitations inherent to meta-analysis methodology. The use of varying diagnostic methods across studies is recognized as a limitation. Additionally, data on specific work practices and hygiene behaviors was limited in the included studies. This limited data availability restricts the ability to definitively assess their impact on infection risk.

## 5. Conclusion

This systematic review and meta-analysis estimated a high global seroprevalence (41%, 95% CI: 36–47%) of *T. gondii* infection among workers occupationally exposed to animals. Substantial heterogeneity was observed, with subgroup analyses revealing significant variations by gender, occupation, geographic region, and income classification. Country-specific factors likely contributed most to this heterogeneity. These findings highlight the need for targeted interventions to reduce the risk of *T. gondii* infection in this high-risk population, considering factors such as occupation and geographic location. Further research is warranted to elucidate the mechanisms underlying the observed disparities and to develop effective preventive strategies.
